# Supplementary material for: Analysis of disaster-affected population mobility through grid-aggregated mobile location data: The 2017 Jiuzhaigou earthquake, China
Source: PLoS One. 2025 Oct 29;20(10):e0335415. doi: 10.1371/journal.pone.0335415 (PMC12571291; doi:10.1371/journal.pone.0335415)
Supplement: Attachment — Submitted filename: PlosONE_Reviewer comments_2025_05_04.pdf [file pone.0335415.s001.pdf]

Dear Authors,

I appreciate the opportunity to review this manuscript, “Analysis of Disaster-Affected Population Mobility Through Grid-Aggregated Mobile Location Data: The 2017 Jiuzhaigou Earthquake, China.” This is a really strong paper with interesting insights on methods to understand population movement after a disaster.

A few overarching comments:

1) The abstract is very clear. However, it does not mention the BBAC_I method which is a substantial part of your results and methods and the conclusion, therefore, does not compare the results across the EOF1, EOF2, and BBAC_I methods. This reviewer recommends enhancing the abstract to reflect all the methods used and the more robust conclusion you reached.
2) When did you access the mobile phone data? Or, more directly, when was the data you accessed available? I appreciate your emphasis in the introduction that the analyses you completed could be implemented quickly after a disaster. However, I do not see a detailed description of the opportunity to complete these analyses soon after the earthquake. Please provide more detail about when the data is available and when you completed the analyses.
3) There are a few results and discussion topics that are not introduced in the methods section. For example, the positive and negative EOF analyses, the Intensity zones, and the BBAC_I clusters are not presented in the methods. Please ensure that you describe in the methods all the ways that you reach the results.
4) Lines 233 – 240, 270 – 274, and 434 - 440 describe interpretations of data that lead beyond the scope of the described study. Please consider removing descriptions of data interpretations that do not directly follow from the analytic methods your study implemented.
5) The discussion presents some results that were not described in the results section. Please ensure all results are mentioned in the results section.

Specific suggestions

1) Lines 94 – 96 describe the structure of a standard scientific paper. Therefore, please consider removing these lines.
2) Lines 109 and 151, at least, references using data from August 2, the day before the earthquake. If the earthquake was on August 8, how is August 2 the day before the earthquake? Please review this detail.
3) Can you please increase the amount of detail you provide about missingness? I understand that some grids have missingness and that you removed those, but I am not fully clear on how much missingness there was. Please share more about what proportion of grids you had to remove.
4) Please consider removing lines 217 and 218 as the sentence describes the conventional purpose of a results section.
5) Line 268 references “the report”. Please ensure that the reader knows exactly what ‘report’ you are referencing.

Thank you very much for this opportunity to review a very interesting paper.
