# Supplementary material for: Analysis of disaster-affected population mobility through grid-aggregated mobile location data: The 2017 Jiuzhaigou earthquake, China
Source: PLoS One. 2025 Oct 29;20(10):e0335415. doi: 10.1371/journal.pone.0335415 (PMC12571291; doi:10.1371/journal.pone.0335415)
Supplement: Attachment — Submitted filename: Response to Reviewers.docx [file pone.0335415.s002.docx]

**Response to Reviewers**

We would like to express our sincere gratitude to the editor and the two reviewers for their constructive feedback on our manuscript. We have carefully considered each of their comments and made substantial revisions throughout the manuscript, including refinements to the figures and language to improve clarity and eliminate potential ambiguities. In addition, the analysis has been further refined where appropriate. All changes are marked in blue in the revised manuscript, which you can see in the additional material. Below are our item-by-item responses.

**Response to Editor**

**Comment 1:** Please ensure that your manuscript meets PLOS ONE's style requirements, including those for file naming.

**Response 1:** Done. We have revised the manuscript to fully comply with PLOS ONE's style requirements.

**Comment 2:** Thank you for stating the following financial disclosure:

This research was supported by the National Key Research and Development Program (2022YFC3004404).

Please state what role the funders took in the study. If the funders had no role, please state: "The funders had no role in study design, data collection and analysis, decision to publish, or preparation of the manuscript."

If this statement is not correct you must amend it as needed.

Please include this amended Role of Funder statement in your cover letter; we will change the online submission form on your behalf.

**Response 2:** Thank you for your guidance. We have added the required statement— “The funders had no role in study design, data collection and analysis, decision to publish, or preparation of the manuscript.”—to our cover letter.

**Comment 3:** We note that you have indicated that there are restrictions to data sharing for this study. For studies involving human research participant data or other sensitive data, we encourage authors to share de-identified or anonymized data. However, when data cannot be publicly shared for ethical reasons, we allow authors to make their data sets available upon request. If there are ethical or legal restrictions on sharing a de-identified data set, please explain them in detail (e.g., data contain potentially identifying or sensitive patient information, data are owned by a third-party organization, etc.) and who has imposed them (e.g., a Research Ethics Committee or Institutional Review Board, etc.). Please also provide contact information for a data access committee, ethics committee, or other institutional body to which data requests may be sent.

**Response 3:** Thank you for your comments. The mobile phone location data used in this study were obtained in 2023 via private communication with Dr. Shen at Beijing Normal University. We are not authorized to share the dataset publicly, as the data are held by Dr. Shen

**Comment 4:** In the online submission form, you indicated that the data underlying the results presented in the study are available from Shishen at Beijing Normal University (Contact E-mail: shens@bnu.edu.cn). This policy applies to all data except where public deposition would breach compliance with the protocol approved by your research ethics board. If your data cannot be made publicly available for ethical or legal reasons (e.g., public availability would compromise patient privacy), please explain your reasons on resubmission and your exemption request will be escalated for approval.

**Response 4:** Thank you for your comments. After discussion with Dr. Shi Shen, we confirm that the mobile phone location data used in this study are subject to legal restrictions and cannot be publicly shared.

The dataset was originally acquired from China Unicom under a data use agreement that includes strict confidentiality clauses. According to the terms of this agreement, Dr. Shen is prohibited from sharing the data without formal authorization from China Unicom. Therefore, the authors do not have the legal right to make the data publicly available.

Interested researchers may request access to the dataset by contacting:

- Dr. Shi Shen (Beijing Normal University)

Email: shens@bnu.edu.cn

- Strategic Customer Department, China Unicom

Phone: +86 18519518331

Access is subject to approval by China Unicom and may require appropriate data use agreements and/or ethical review, depending on the nature of the request.

**Comment 5:** Please amend your list of authors on the manuscript to ensure that each author is linked to an affiliation. Authors’ affiliations should reflect the institution where the work was done.

**Response 5:** Done. Please see the details in the revised manuscript on Page 1, Lines 4-10 as follows.

*“*

*Zezhi Lin^2^, Rui Mao^1,*^, Huaiqun Zhao^1^, Zihui Tang^3^, Saini Yang^1^, Po Pan^4^*

*^1^ School of National Safety and Emergency Management, Beijing Normal University, Beijing, 100875, China*

*^2^ School of Systems Science, Beijing Normal University, Beijing, 100875, China*

*^3^ School of Architecture and Urban Planning, Beijing University of Civil Engineering and Architecture, Beijing, 102627, China*

*^4^ Unicom (Beijing) Industry Internet Co.,Ltd., Beijing, 100038, China*

** Corresponding author*

*”*

**Comment 6:** We note that Figure 1, 3, 4, 5, in your submission contain [map/satellite] images which may be copyrighted. All PLOS content is published under the Creative Commons Attribution License (CC BY 4.0), which means that the manuscript, images, and Supporting Information files will be freely available online, and any third party is permitted to access, download, copy, distribute, and use these materials in any way, even commercially, with proper attribution. For these reasons, we cannot publish previously copyrighted maps or satellite images created using proprietary data, such as Google software (Google Maps, Street View, and Earth). For more information, see our copyright guidelines: http://journals.plos.org/plosone/s/licenses-and-copyright.

**Response 6:** Thank you for your comments. The base maps used in Figures 1, 3, 4, and 5 of our manuscript were obtained from the National Platform for Common GeoSpatial Information Services (https://www.tianditu.gov.cn/).

This platform is a government-operated service in China that provides publicly available geospatial data and base maps for academic, educational, and non-commercial purposes. To the best of our knowledge, the use of these maps is permitted under open government data policies, and they are not subject to commercial copyright restrictions like Google Maps or similar proprietary services. Please see the details in the “map_copyright_statement” file, we have uploaded it as an "Other" file with our submission.

**Comment 7:** Please review your reference list to ensure that it is complete and correct. If you have cited papers that have been retracted, please include the rationale for doing so in the manuscript text, or remove these references and replace them with relevant current references. Any changes to the reference list should be mentioned in the rebuttal letter that accompanies your revised manuscript. If you need to cite a retracted article, indicate the article’s retracted status in the References list and also include a citation and full reference for the retraction notice.

**Response 7:** Thank you for your comments. All in-text citations and references have been thoroughly checked and corrected to comply with the PLOS ONE formatting guidelines. Please see the details in the revised manuscript on Pages 28-31, Lines 508-616 as follows.

*“*

*1.	Butuzova A, Dolgikh V. Study of the target structure of transport mobility of the population on the example of Irkutsk. Transport Technician: Education and Practice. 2023 Sep 9;4:322–31.*

*2.	Opdyke A, Lepropre F, Javernick-Will A, Koschmann M. Inter-organizational resource coordination in post-disaster infrastructure recovery. Construction Management and Economics. 2017 Sep 2;35(8–9):514–30.*

*3.	Hu L, Fang Z, Zhang M, Jiang L, Yue P. Facilitating Typhoon-Triggered Flood Disaster-Ready Information Delivery Using SDI Services Approach—A Case Study in Hainan. Remote Sensing. 2022 Jan;14(8):1832.*

*4.	Liu C, Chen Y, Wei Y, Chen F. Spatial Population Distribution Data Disaggregation Based on SDGSAT-1 Nighttime Light and Land Use Data Using Guilin, China, as an Example. Remote Sensing. 2023 Jun 3;15(11):2926.*

*5.	Li J, He Z, Plaza J, Li S, Chen J, Wu H, et al. Social Media: New Perspectives to Improve Remote Sensing for Emergency Response. Proceedings of the IEEE. 2017 Oct;105(10):1900–12.*

*6.	Yao K, Yang S, Tang J. Rapid assessment of seismic intensity based on Sina Weibo — A case study of the changning earthquake in Sichuan Province, China. Int J Disaster Risk Reduct. 2021 May;58:102217.*

*7.	Kawade SS, Akant K. Real Time Image Processing System for Crop Segmentation. In: 2021 6th International Conference for Convergence in Technology (I2CT); 2021. p. 1–6. doi: 10.1109/I2CT51068.2021.9417921.*

*8.	Lu Z, Long Z, Xia J, An C. A Random Forest Model for Travel Mode Identification Based on Mobile Phone Signaling Data. Sustainability. 2019 Oct 25;11(21):5950.*

*9.	Yabe T, Jones NKW, Rao PSC, Gonzalez MC, Ukkusuri SV. Mobile phone location data for disasters: A review from natural hazards and epidemics. Computers, Environment and Urban Systems. 2022 Jun;94:101777.*

*10.	Yabe T, Tsubouchi K, Sudo A, Sekimoto Y. A framework for evacuation hotspot detection after large scale disasters using location data from smartphones: case study of Kumamoto earthquake. In: Proceedings of the 24th ACM SIGSPATIAL International Conference on Advances in Geographic Information Systems; 2016. p. 44. doi: 10.1145/2996913.2997014.*

*11.	Wilson R, zu Erbach-Schoenberg E, Albert M, Power D, Tudge S, Gonzalez M, Guthrie S, Chamberlain H, Brooks C, Hughes C, Pitonakova L. Rapid and near real-time assessments of population displacement using mobile phone data following disasters: The 2015 Nepal earthquake. PLoS currents. 2016 Feb 24;8:ecurrents-dis.*

*12.	Duan Z, Lei Z, Zhang M, Li W, Fang J, Li J. Understanding evacuation and impact of a metro collision on ridership using large-scale mobile phone data. IET Intelligent Transport Systems. 2017;11(8):511–20.*

*13.	Xia C, Nie G, Fan X, Zhou J, Pang X. Research on the application of mobile phone location signal data in earthquake emergency work: A case study of Jiuzhaigou earthquake. Martínez-Álvarez F, editor. PLOS ONE. 2019 Apr 12;14(4):e0215361.*

*14.	Acosta RJ, Kishore N, Irizarry RA, Buckee CO. Quantifying the dynamics of migration after Hurricane Maria in Puerto Rico. Proc Natl Acad Sci. 2020 Dec 22;117(51):32772–8.*

*15.	Tan S, Lai S, Fang F, Cao Z, Sai B, Song B, et al. Mobility in China, 2020: a tale of four phases. Natl Sci Rev. 2021 Dec 4;8(11):nwab148.*

*16.	Guo X, Wei B, Nie G, Su G. Application of Mobile Signaling Data in Determining the Seismic Influence Field: A Case Study of the 2017 Mw 6.5 Jiuzhaigou Earthquake, China. International Journal of Environmental Research and Public Health. 2022 Jan;19(17):10697.*

*17.	Wei B, Su G, Liu F. Dynamic Assessment of Spatiotemporal Population Distribution Based on Mobile Phone Data: A Case Study in Xining City, China. Int J Disaster Risk Sci. 2023;14:649–665. doi: 10.1007/s13753-023-00480-3.*

*18.	Yabe T, Sekimoto Y, Tsubouchi K, Ikemoto S (2019) Cross-comparative analysis of evacuation behavior after earthquakes using mobile phone data. PLoS ONE 14(2): e0211375. https://doi.org/10.1371/journal.pone.0211375.*

*19.	Hong B, Bonczak BJ, Gupta A, Kontokosta CE. Measuring inequality in community resilience to natural disasters using large-scale mobility data. Nat Commun. 2021 Mar 25;12(1):1870.*

*20.	Deng H, Aldrich DP, Danziger MM, Gao J, Phillips NE, Cornelius SP, et al. High-resolution human mobility data reveal race and wealth disparities in disaster evacuation patterns. Humanit Soc Sci Commun. 2021 Jun 15;8(1):1–8.*

*21.	Rahimi-Golkhandan A, Garvin MJ, Wang Q. Assessing the Impact of Transportation Diversity on Postdisaster Intraurban Mobility. Journal of Management in Engineering. 2021 Jan 1;37(1):04020106.*

*22.	Yabe T, Ukkusuri SV, C. Rao PS. Mobile phone data reveals the importance of pre-disaster inter-city social ties for recovery after Hurricane Maria. Appl Netw Sci. 2019 Dec;4(1):98.*

*23.	Bengtsson L, Lu X, Thorson A, Garfield R, Von Schreeb J. Improved Response to Disasters and Outbreaks by Tracking Population Movements with Mobile Phone Network Data: A Post-Earthquake Geospatial Study in Haiti. Gething PW, editor. Plos Med. 2011 Aug 30;8(8):e1001083.*

*24.	Podesta C, Coleman N, Esmalian A, Yuan F, Mostafavi A. Quantifying community resilience based on fluctuations in visits to points-of-interest derived from digital trace data. Journal of The Royal Society Interface. 2021 Apr;18(177):20210158.*

*25.	Cheng Y, Church G. Biclustering of Expression Data. Proceedings / . International Conference on Intelligent Systems for Molecular Biology ; ISMB International Conference on Intelligent Systems for Molecular Biology. 2000 Feb 1;8:93–103.*

*26.	Dai K, Cheng C, Shen S, Su K, Zheng X, Zhang T. Postearthquake situational awareness based on mobile phone signaling data: An example from the 2017 Jiuzhaigou earthquake. Int J Disaster Risk Reduct. 2022 Feb;69:102736.*

*27.	The Chinese Seismic Intensity Scale. Standards Press of China, Beijing, China; 2020. Available from: https://kns.cnki.net/kcms2/article/abstract?v=-93ivAxQXRqFgn1DcmW2S-9m_QCqsjgrPKcoKZn4pezR3ebCWzCPkcM9WBYLyyY9rhS6aT9nhcK0VBXd2zFTxsi44A_x4zRi41R-iaKEvL8FEVC5Iqo3WZIqIXGwtb8u80S8Bk6zHEpTr6iE58RZQQ==&uniplatform=NZKPT&language=CHS*

*28.	Li S, Chen Y, Yu T. Comparison of macroseismic-intensity scales by considering empirical observations of structural seismic damage. Earthquake Spectra. 2021 Feb 1;37(1):449–85.*

*29.	Hannachi A, Jolliffe IT, Stephenson DB. Empirical orthogonal functions and related techniques in atmospheric science: A review. International Journal of Climatology. 2007;27(9):1119–52.*

*30.	Monahan AH, Fyfe JC, Ambaum MHP, Stephenson DB, North GR. Empirical Orthogonal Functions: The Medium is the Message. Journal of Climate. 2009 Dec 15;22(24):6501–14.*

*31.	Wilks DS. Principal Component (EOF) Analysis. In: Wilks DS, editor. Statistical Methods in the Atmospheric Sciences. 4th ed. Amsterdam: Elsevier; 2019. p. 617–668. doi: 10.1016/B978-0-12-815823-4.00013-4.*

*32.	Wu X, Poorthuis A, Zurita-Milla R, Kraak MJ. An interactive web-based geovisual analytics platform for co-clustering spatio-temporal data. Computers & Geosciences. 2020 Apr 1;137:104420.*

*33.	Banerjee A, Dhillon I, Ghosh J, Merugu S, Modha DS. A generalized maximum entropy approach to bregman co-clustering and matrix approximation. In: Proceedings of the Tenth ACM SIGKDD International Conference on Knowledge Discovery and Data Mining; 2004. p. 509–514. doi: 10.1145/1014052.1014111.*

*34.	Wu X, Zurita-Milla R, Kraak MJ. Co-clustering geo-referenced time series: exploring spatio-temporal patterns in Dutch temperature data. Int J Geogr Inf Sci. 2015 Apr 3;29(4):624–42.*

*35.	Wu X, Cheng C, Qiao C, Song C. Spatio-temporal differentiation of spring phenology in China driven by temperatures and photoperiod from 1979 to 2018. Science China Earth Sciences. 2020 Oct 1;63(10):1485–98.*

*36.	Nattino F, Ku O, Grootes MW, Izquierdo-Verdiguier E, Girgin S, Zurita-Milla R. CGC: a Scalable Python Package for Co- andTri-Clustering of Geodata Cubes. Journal of Open Source Software. 2022 Apr 10;7(72):4032.*

*37.	Chen X. Experience and Lessons of Public Evacuation After the “8·8” JiuZhaigou Earthquake in Sichuan Province. Journal of China Emergency Management Science. 2019;(36):36–43.*

*38.	Zhang X, Lu Q, Ning B, Huang Z. A rapid identification method for severely earthquake-damaged areas based on damaged mobile phone base stations in China. Environ Earth Sci. 2016 Apr 13;75(8):704.*

*”*

**Response to Reviewer #1**

**Comment 1:** It is recommended to add an analytical framework figure and a location map of the study area, to clarify the spatial context of the analysis.

**Response 1:** Thank you for your comments. To enhance clarity, we have added an analytical framework (Fig. 1) and a study area location map (Fig. 2) to illustrate the methodology and spatial setting. Please refer to Figs. 1 and 2 below for details.

Fig. 1 Analytical framework of the study

Fig. 2 Spatial intensity distribution on August 9, 2017

**Comment 2:** In the introduction, the fourth paragraph presents the advantages of the EOF method and states its use in this study, while the beginning of the fifth paragraph points out the shortcomings of the current method, BBAC_I. It is suggested that the authors first discuss the deficiencies of the currently adopted method, and then summarize the advantages of the EOF method and explain why it was chosen.

**Response 2:** Thank you for your insightful comments. We have revised the structure of the Introduction to first discuss the strengths and limitations of the current method (BBAC_I), and then introduce the advantages of the EOF method and the reason for its adoption. Please see the details in the revised manuscript on Pages 4-5, Lines 78-101 as follows.

*“To address this issue, researchers have explored various analytical methods that aim to extract meaningful information from grid-level data. Among these, the Bregman Block Average Co-clustering algorithm with I-divergence (BBAC_I) has emerged as an efficient spatiotemporal clustering technique [25] and has been applied to analyze population changes during disaster events, including the 2017 Jiuzhaigou earthquake [26]. BBAC_I is capable of identifying regions with similar temporal trends in population variation. However, it has limited ability to identify critical impact zones or uncover directional movement flows—especially when applied to anonymized, gridded datasets without trajectory data.*

*To overcome these limitations, this study explores the use of the Empirical Orthogonal Function (EOF) method, which decomposes spatial-temporal data into orthogonal modes that reflect dominant patterns of variation. By detecting positive and negative anomalies in EOF components, this method allows for the exploration of population changes over time, enabling preliminary assessments of movement directions and intensities during disasters. For example, during earthquakes, populations often evacuate from high-risk areas to safer regions, resulting in population increases in shelters, decreases in hazardous zones, and fluctuations along evacuation routes. By analyzing these population spatial changes with EOF analysis, it becomes possible to infer evacuation directions and patterns even in the absence of individual trajectory data.*

*Since both the EOF and BBAC_I methods are capable of capturing spatial-temporal population changes, this study applies both approaches to analyze population movements following the 2017 Jiuzhaigou earthquake. Their results were compared to evaluate the strengths and limitations of each approach in capturing post-disaster population dynamics.”*

**Comment 3:** In Section 2.2.1(1), regarding 'Eliminate grids with three or more consecutive missing values in their time series', the grids that did not capture data did so for unknown reasons. Could these unknown reasons affect the study’s results? It is recommended to include a spatial distribution map of the data to more clearly demonstrate its distribution. And visualize the spatial distribution of missing grids to assess whether their exclusion introduces regional bias.

**Response 3:** Thank you for your insightful comments. We have added a figure (Fig. 2) to illustrate the spatial distribution of grid validity, distinguishing between valid grids and those with three or more consecutive missing values (invalid grids). Of the 683 total grid cells, approximately 46% were classified as valid, while the remaining 54% were considered invalid. Although invalid grids were more numerous, they accounted for only 13.8% of the total population on August 2. Since both August 2 and August 9 were Wednesdays, population patterns are expected to be similar. Given the low pre-earthquake population in invalid grids, it is reasonable to assume that their post-earthquake population was also limited. Moreover, Fig. 2 shows that valid and invalid grids exhibit similar geographic distribution patterns. These findings support the conclusion that excluding invalid grids is unlikely to introduce significant spatial or demographic bias. Please see the details in the revised manuscript on Page 9, Lines 165-175 as follows.

*“**Prior to data processing, we identified 683 grid cells with mobile location data available for at least one timestamp. Among these, approximately 46% were classified as valid grids after the above data procession steps (i.e., with less than three consecutive missing values in their time series), while the remaining 54% were considered invalid. Although invalid grids were more numerous, they accounted for only 13.8% of the total population on August 2. Since both August 2 and August 9 were Wednesdays, population patterns are expected to be similar.* *Given the low pre-earthquake population in invalid grids,* *it is reasonable to assume that their post-earthquake population was also limited. Moreover, Fig 2 shows that valid and invalid grids exhibit similar geographic distribution patterns. These findings support the conclusion that excluding invalid grids is unlikely to introduce significant spatial or demographic bias.”*

**Comment 4:** The manuscript states, 'After removing these grids, we performed an inner join on the remaining grids to ensure that only spatial regions with comparable data before and after the disaster were included in the analysis.' What exactly is meant by 'inner join' in this context?

**Response 4:** Thank you for your comments. To avoid potential confusion, we have removed the term “inner join” and revised the sentence for greater clarity. The revised text now explains that we retained grid cells with data for both observation dates—August 2, 2017 (six days before the earthquake) and August 9, 2017 (the day after the earthquake). This ensures a consistent spatial scope for analysis and improves the accuracy of assessing the earthquake’s impact on population. Please see the details in the revised manuscript on Page 8, Lines 143-149 as follows.

*“**After this filtering step, we retained grid cells with data for both observation dates: August 2, 2017 (six days before the earthquake) and August 9, 2017 (the day after the earthquake). This ensured consistency in the spatial scope of the analysis and improved the accuracy of assessing the earthquake's impact on population. Based on this filtered dataset, we calculated the difference in population counts between the two dates for each grid to generate population anomaly data for further analysis.”*

**Comment 5:** In Fig 1, 'Spatial intensity distribution on August 9, 2017', is the intensity describing the grid cells? It is recommended to add the official intensity map for comparison. Moreover, the figure does not clearly show the comparison between the anomaly data and the macroseismic intensity. Currently, the figure’s caption is ambiguous. It is unclear whether the colors represent the actual seismic intensity levels or the magnitudes of the data-derived anomalies.

**Response 5:** Thank you for your insightful comments. The Fig 1 in the original manuscript has been revised and is now presented as Fig. 2 in this reply. In this new figure, we use the official seismic intensity map as the base layer, with macroseismic intensity shown through concentric intensity contours. This clarifies that the term “intensity” refers to official macroseismic measurements rather than data-derived anomalies. We have also overlaid the spatial distribution of grids, where grid colors indicate data validity, not seismic intensity, to improve interpretability. Additionally, the term “anomaly data” was a misstatement and has been corrected to “valid grids” in the caption of Fig. 2.

**Comment 6:** It is suggested to merge the infrastructure POIs shown in Fig 5 into Fig 3(a) and (b) to reduce the number of figures.

**Response 6:** Done. Please see Fig. 3 in this reply for the details.

Fig. 3 The EOF analysis results on August 9

**Comment 7:** “In particular, regions with an earthquake intensity below VI generally do not suffer destructive seismic impacts and are not typically considered in seismic fortification (Nazmfar et al. 2019; Li et al. 2021). Therefore, our study area encompasses regions where the seismic intensity is above V, representing areas at greater risk of damage”. Does the seismic intensity above V represents areas at greater risk of damage?

**Response 7:** Thank you for your comment. We agree that the original statement “representing areas at greater risk of damage” may have been overly definitive, and we have removed it to avoid potential misinterpretation. In this study, we follow the Chinese seismic intensity standard, which considers regions with intensity below VI to be at low risk for destructive seismic impacts. Therefore, we focus on areas with intensity of VI and above, where the population is more likely to be affected by earthquake damage. Please see the revised sentence in the updated manuscript on Page 9, Lines 181-183 as follows.

*“Based on this standard, our study focuses on areas where the seismic intensity is VI or above, where the population is more likely to be exposed to potential earthquake impacts.”*

**Comment 8:** Language and writing need to be further improved.

**Response 8:** Done. We invited a native speaker to carefully proofread the entire manuscript, ensuring improvements in grammar, clarity, and logical coherence.

**Comment 9:** The format of the reference citation in the main text is not standard, please revise them.

**Response 9:** Done. All in-text citations and references have been thoroughly checked and corrected to comply with the PLOS ONE formatting guidelines.

**Response to Reviewer #2**

**Comment 1:** The abstract is very clear. However, it does not mention the BBAC_I method which is a substantial part of your results and methods and the conclusion, therefore, does not compare the results across the EOF1, EOF2, and BBAC_I methods. This reviewer recommends enhancing the abstract to reflect all the methods used and the more robust conclusion you reached.

**Response 1:** Thank you for your insightful comments. We have rewritten our abstract to include the results across the EOF1, EOF2, and BBAC_I methods, highlighting their respective contributions to the results. Please see the updated abstract in the revised manuscript on Pages 2-3, Lines 21-45 as follows.

*“**In disaster research, individual-level mobile phone location data is considered highly valuable for assessing population mobility and disaster impacts. However, due to privacy regulations in China, only spatially aggregated mobile data with a resolution of 1 km × 1 km are available. These data do not contain explicit population individual population movement, which poses challenges for analyzing population movement patterns in disaster research. To using this grid-based mobile data to describe population movement, we applied an empirical orthogonal function (EOF) method to the post-disaster phase of the 2017 Jiuzhaigou earthquake. The first EOF mode (EOF1) primarily exhibits positive anomalies centered over the Jiuzhaigou Valley. The principal components for the EOF1 show a decreasing trend from midnight to 20:00, indicating a continuous outflow of population from the Jiuzhaigou Valley during this period. The second mode (EOF2) exhibits negative anomalies at the Jiuzhaigou Valley and along the road to the southwest of the Valley, while positive anomalies appear along two roads, i.e., one extending from the Jiuzhaigou Valley to Shuanghe, and the other from the Chuanzhusi Town government square to western Chuanzhusi. The primary components of EOF2 reveal that, from midnight to 10:00, population increased along these two roads while decreasing over the Jiuzhaigou Valley and the road leading southward to the Chuanzhusi Town government square. After 10:00, this population change pattern diminished between 10:00 to 15:00. Based on the EOF2 results, two evacuation routes were identified: Path 1 extended northwest from the Chuanzhusi Town government square; Path 2 led southeast from Jiuzhaigou Valley through Shuanghe Town. In comparison, the BBAC_I clustering method identifies clusters with similar temporal trends but fails to pinpoint the most affected areas or infer evacuation directions. In contrast, EOF analysis overcomes these limitations by revealing key impact zones and evacuation patterns, even in the absence of trajectory data.”*

**Comment 2:** When did you access the mobile phone data? Or, more directly, when was the data you accessed available? I appreciate your emphasis in the introduction that the analyses you completed could be implemented quickly after a disaster. However, I do not see a detailed description of the opportunity to complete these analyses soon after the earthquake. Please provide more detail about when the data is available and when you completed the analyses.

**Response 2:** Thank you for your comments. The mobile phone location data used in this study were obtained in 2023 via private communication with Dr. Shen at Beijing Normal University (Contact: shens@bnu.edu.cn). We have described the dataset in the revised manuscript on Page 6, Lines 113–117, and acknowledged Dr. Shen as the data provider in the Acknowledgements section (Page 27, Lines 513–514). In addition, the dataset was originally used in the following study:

*Dai, K., Cheng, C., Shen, S., Su, K., Zheng, X., & Zhang, T. (2022). Postearthquake situational awareness based on mobile phone signaling data: An example from the 2017 Jiuzhaigou earthquake. International Journal of Disaster Risk Reduction, 69, 102736.* [*https://doi.org/10.1016/j.ijdrr.2021.102736*](https://doi.org/10.1016/j.ijdrr.2021.102736)

**Comment 3:** There are a few results and discussion topics that are not introduced in the methods section. For example, the positive and negative EOF analyses, the Intensity zones, and the BBAC_I clusters are not presented in the methods. Please ensure that you describe in the methods all the ways that you reach the results.

**Response 3:** Thank you for your insightful comments. The background information and analytical procedures are clarified as follows.

(1) Regarding data sources:

The seismic intensity zones used in our study are derived from the official post-earthquake intensity map released by the China Earthquake Administration. Since the intensity levels in each region are determined externally and not calculated within our study, this map serves solely as background information to contextualize the study area. It is not part of our analytical methodology.

(2) Regarding methodological additions:

We have added a description of the positive and negative phases in the EOF (Empirical Orthogonal Function) analysis to the Methods section to clarify how these modes are interpreted in the results. We have also included a brief mathematical explanation of the BBAC_I clustering process to clarify its iterative procedure.

Please see the details in the revised manuscript on Pages 10-11, Lines 202-209 and Pages 13-14, Lines 248-254 as follows.

*“*

*(EOF Page 11, Lines 205-213)*

*Interpretation of EOF results involves examining both the spatial and temporal components. In the spatial modes, positive and negative values indicate grids where the variable (i.e., population count) deviates from the mean in opposite directions, indicating spatial patterns of population inflow or outflow. While the signs of the EOFs and PCs are mathematically arbitrary—multiplying both by −1 does not alter the result—their physical interpretation depends on their combination. For example, a positive PC value associated with a positive EOF anomaly suggests population increase in those grids, whereas a negative EOF anomaly would suggest a decrease. The interpretation reverses when the PC is negative.*

*(BBAC_I Pages 13-14, Lines 243-249)*

*The optimization objective of BBAC_I is to minimize the information divergence between the original and co-clustered matrices. The corresponding loss function is defined as the following formula:*

$F_{loss}= D_{I} (O(G,T) || \hat{O}(\hat{G},T))$	*(2)*

*where* $D_{I}$ *denotes the I-divergence, a form of generalized Bregman divergence. Through iterative updates of cluster assignments, the algorithm seeks to minimize* $F_{loss}$*, thereby enhancing intra-cluster similarity and inter-cluster differentiation.*

*”*

**Comment 4:** Lines 233 - 240, 270 - 274, and 434 - 440 describe interpretations of data that lead beyond the scope of the described study. Please consider removing descriptions of data interpretations that do not directly follow from the analytic methods your study implemented.

**Response 4:** Done.

**Comment 5:** The discussion presents some results that were not described in the results section. Please ensure all results are mentioned in the results section.

**Response 5:** Thank you for your comments. We have restructured the manuscript to ensure that all results discussed are presented in the Results section. Specifically, the original Section 4.1 has been moved to become Section 3.3, and Section 4.3 is now renumbered as Section 4.1. In addition, the original Section 4.2 has been integrated into the EOF analysis section (Section 3.2). The corresponding revisions can be found in Section 3.2 of the revised manuscript (Page 21, Lines 374–382) as follows.

*“To further validate the inferred spatial patterns, we mapped real-world points of interest (POIs) identified from evacuation reports and media sources such as People’s Daily, Sichuan Daily onto the EOF2 anomaly map (Fig 4B). Major evacuation hubs—such as Chuanzhusi Government Square, Jiuzhaigou Huanglong Airport and hotels distributed throughout Jiuzhaigou Valley—were located within areas of strong negative anomalies and served as initial gathering sites. Conversely, sites near zones of strong positive anomalies, such as the Baihe County and Jiuzhaigou County government buildings, functioned as transit or secondary shelter sites. These correspondences reinforce the robustness of the EOF2-derived evacuation interpretations.”*

**Comment 6:** Lines 94 - 96 describe the structure of a standard scientific paper. Therefore, please consider removing these lines.

**Response 6:** Done.

**Comment 7:** Lines 109 and 151, at least, references using data from August 2, the day before the earthquake. If the earthquake was on August 8, how is August 2 the day before the earthquake? Please review this detail.

**Response 7:** Thank you for your comments. This was a misstatement in the original text. We have corrected the description to indicate that August 2 is six days before the earthquake.

**Comment 8:** Can you please increase the amount of detail you provide about missingness? I understand that some grids have missingness and that you removed those, but I am not fully clear on how much missingness there was. Please share more about what proportion of grids you had to remove.

**Response 8:** Thank you for your insightful comments. We have added a figure (Fig. 2) to illustrate the spatial distribution of grid validity. Of the 683 total grid cells, approximately 46% were classified as valid, while the remaining 54% were considered invalid. Therefore, a total of 368 grids were excluded from the dataset due to exceeding the missingness threshold. Although invalid grids were more numerous, they accounted for only 13.8% of the total population on August 2. Since both August 2 and August 9 were Wednesdays, population patterns are expected to be similar. Given the low pre-earthquake population in invalid grids, it is reasonable to assume that their post-earthquake population was also limited. Moreover, Fig. 2 shows that valid and invalid grids exhibit similar geographic distribution patterns. These findings support the conclusion that excluding invalid grids is unlikely to introduce significant spatial or demographic bias. Please see the details in the revised manuscript on Page 9, Lines 165-175 as follows.

*“Prior to data processing, we identified 683 grid cells with mobile location data available for at least one timestamp. Among these, approximately 46% were classified as valid grids after the above data procession steps (i.e., with less than three consecutive missing values in their time series), while the remaining 54% were considered invalid. Although invalid grids were more numerous, they accounted for only 13.8% of the total population on August 2. Since both August 2 and August 9 were Wednesdays, population patterns are expected to be similar. Given the low pre-earthquake population in invalid grids, it is reasonable to assume that their post-earthquake population was also limited. Moreover, Fig 2 shows that valid and invalid grids exhibit similar geographic distribution patterns. These findings support the conclusion that excluding invalid grids is unlikely to introduce significant spatial or demographic bias.”*

**Comment 9:** Please consider removing lines 217 and 218 as the sentence describes the conventional purpose of a results section.

**Response 9:** Done.

**Comment 10:** Line 268 references “the report”. Please ensure that the reader knows exactly what ‘report’ you are referencing.

**Response 10:** Thank you for your comment. We have added a specific citation for the report referenced in Lines 268, 307, as well as in other places where conclusions from the report are used. We have also clarified that it is an official post-disaster relief report. For example, the revised manuscript now includes the following sentence on Page 17, Lines 307–309:

*“These observed patterns align with the official rescue report [1], which documented several hotels served as temporary shelters in Intensity VIII and IX zones and early rescue operations in the Jiuzhaigou Scenic Area starting at midnight on August 9.”*

Reference

1. Chen X. Experience and Lessons of Public Evacuation After the “8·8” JiuZhaigou Earthquake in Sichuan Province. Journal of China Emergency Management Science. 2019; 36–43.
